# Recent advancements in the use of exosomes as drug delivery systems

**DOI:** 10.1186/s12951-018-0403-9

**Published:** 2018-10-16

**Authors:** Edwin J. Bunggulawa, Wei Wang, Tieying Yin, Nan Wang, Colm Durkan, Yazhou Wang, Guixue Wang

**Affiliations:** 1 Key Laboratory of Biorheological Science and Technology, Ministry of Education, State and Local Joint Engineering Laboratory for Vascular Implants, College of Bioengineering, Chongqing University, No 174 Shazheng Street, Shapingba District, Chongqing, 400044 People’s Republic of China; 2 Nanoscience Centre, Department of Engineering, University of Cambridge, JJ Thomson Avenue, Cambridge, CB3 0FF UK

**Keywords:** Extracellular vesicles, Exosome, Delivery properties

## Abstract

Extracellular vesicles (EVs) are the substances that are released by most types of cells and have an important role in cell to cell communication. Among the most highly researched EVs are exosome. Recent studies show that exosomes derived from cells have different roles and targets. Many studies show that exosome can efficiently deliver many different kinds of cargo to the target cell. Therefore, they are often used to deliver therapeutic cargo for treatment. The exosomes that have been used include both natural ones and those that have been modified with other substances to increase the delivery ability. This article provides a review of both exosomes derived from various cells and modified exosome and their ability in delivering the many kinds of cargo to the target cell.

## Background

Cells communicate with each other primarily through the use of chemical messengers. These are predominantly in the form of extracellular vesicles (EVs). Much recent works has focus on EVs for therapeutic medicine development due to their unique structure which means that they can be modified to contain specific proteins, genetic lipids, and genetic materials including messenger RNA (mRNA), microRNA (mRNA), and other small non-coding RNAs, and genomic DNA (gDNA) from their progenitor cell. Based on an EVs’ origin and size, they can be divided into three groups: (a) exosome (diameter in the range 30–150 nm), (b) microvesicles or ectosomes (50 nm–1 µm), and (c) apoptotic body (50 nm–5 µm) [1]. Due to their nanoscale dimensions, exosomes are considered to be the most promising as a tool for drug delivery to targeted organs, hence they have received the most research attention in recent years.

The exosome is derived from a multivesicular body (MVB) when the MVB fuses with the plasma membrane and is released into the extracellular environment. Its role is as a communication tool to deliver a chemical cargo to the recipient cell [2]. Exosome also plays a role in intercellular communication by carrying proteins and RNAs between neighboring cells or even to distant organs [3]. Exosomes bind to cell membranes through receptor–ligand interaction and mediate antigen presentation, cancer progression etc. Exosomes deliver their surface protein and cytoplasm to the recipient cell by fusing with the target-cell membrane at which point they open up. Many studies show that exosomes enter cells, release their cargo and mediate many physiological and pathological processes [4].

Exosomes can contain many types of biomolecule, including proteins, carbohydrates, lipids and nucleic acids. This varies depending on the EV’s origin, its physiological and pathological state, and even the precise cellular release site. The protein composition within can also mark the existence of disease pathologies such as cancer or inflammatory diseases; however, exosomes also contain a number of common proteins as well as those that participate in vesicle formation and trafficking [5].

Almost all bodily fluids contain exosomes that contain structures formed within every cell type. These structures/contents are often disease-specific such as in viral infections, neurodegenerative diseases (prions, Alzheimer, Huntington disease), and cancer. As a result, exosomes are being intensively investigated as a source of novel biomarkers. Numerous studies have been focus on understanding the function of exosomes in mediating intercellular communication, immune system functions, development and differentiation, neuronal function, cell signaling, regeneration, and several of the steps in viral replication [6]. The discovery of the role of exosomes has led the researchers to explore their use as delivery vehicles of a variety of medicines. As exosomes are naturally-formed and are involved in many biological and pathological processes, they have multiple advantages when compared to other nanoparticles.

### Biogenesis of exosomes

**Fig. 1:**
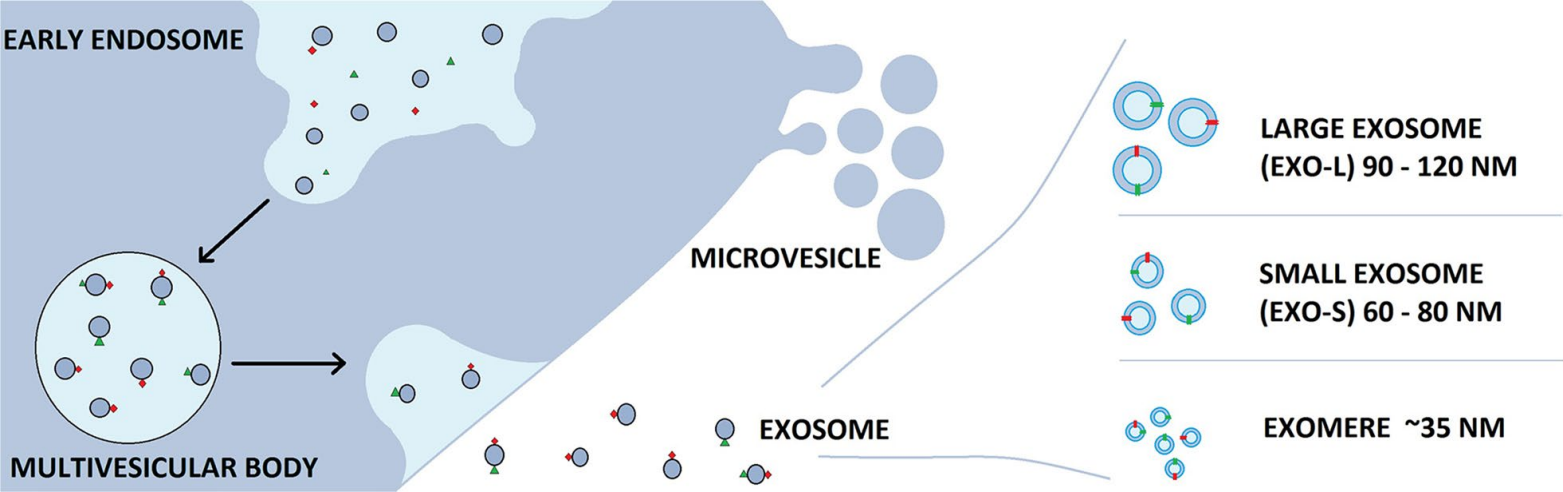
Biogenesis of exosome

Biogenesis of exosomes starts in the endosomal system (Fig. 1). The process starts from early endosomes that mature to became late endosomes or multivesicular bodies, which the endosomal membrane invaginates to produce intraluminal vesicles (ILVs) in the lumen of organelles [7]. The MVBs fuse with the plasma membrane of the cell and release ILVs (which we refer to as exosomes) into the extracellular environment in an exocytotic fashion. They can be secreted under both normal and pathological conditions by a wide array of cell types [8]. Fusion of MVBs with the cell membrane and spatiotemporal traffic of vesicles are regulated by Rab GTPase. Endosomal-sorting complex required for transport (ESCRT) is the central molecular machinery of exosome formation at endosomes [9].

Both ESCRT-dependent and independent signals have been suggested as determining the sorting of exosomes. Syndecan heparin sulfate proteoglycans and their cytoplasmic adaptor syntenin have been found to control the formation of exosomes. Many processes have been proposed as mechanisms that mediate the uptake of exosomes, such as fusion with cellular membrane of the recipient cell, juxtacrine signaling through receptor-ligand interactions and endocytosis by phagocytosis. There are several proteins that are suspected as potential receptor for exosome uptakes, such as Tim1/4 for B cells and ICAM-1 for APCs. However, the specific receptors still remain unclear [10].

In 2018, Zhang et al. further developed the potential for the use of exosomes. They used asymmetric flow field-flow fractionation (AF4) technology and found two exosome subpopulations that they termed, large exosomes (exo-L) and small exosomes (exo-S) and identified a distinct nanoparticle type they termed “exomere”. Exo-L (90–120 nm), Exo-S (60–80 nm) and exomeres (~ 35 nm) have different characteristic and properties and also have different roles. This research has opened an avenue for studies related to exosomes for diagnostic, prognostic and therapeutic applications [11].

#### Exosome cellular recognition

Many studies have been carried out on exosome biogenesis, including cellular recognition and internalization of exosomes. Cellular recognition of exosomes can be divided into three ways; free floating, adhesion, and antigen recognition. Exosome cellular recognition by free floating is similar to that of liposomes which could be mediated by opsonization of exosomes during circulation. Exosomes use chemokines to attract leukocytes to their location. Exosomes express an impressive array of chemokines, which may attract T cells and other cell types.

Exosome cellular recognition by adhesion is a fundamental step for communication between exosomes and T cells. Adhesion by exosomes requires the conformational change of integrins from a low to a high affinity status which enables oligomerization of integrins and coupling with cytoskeletal elements, to facilitate the high avidity binding of lymphocyte to the integrin-bound exosomes.

For the case of cellular recognition by antigen recognition, molecular profiling and proteomic analysis show that target cell specificity for exosomes is dictated by a combination of antigen and MHC class I and II molecules. MHC’s expressions on exosomes are dependent on the expression of these molecules on the parent cell [12].

#### Exosome uptake

**Fig. 2:**
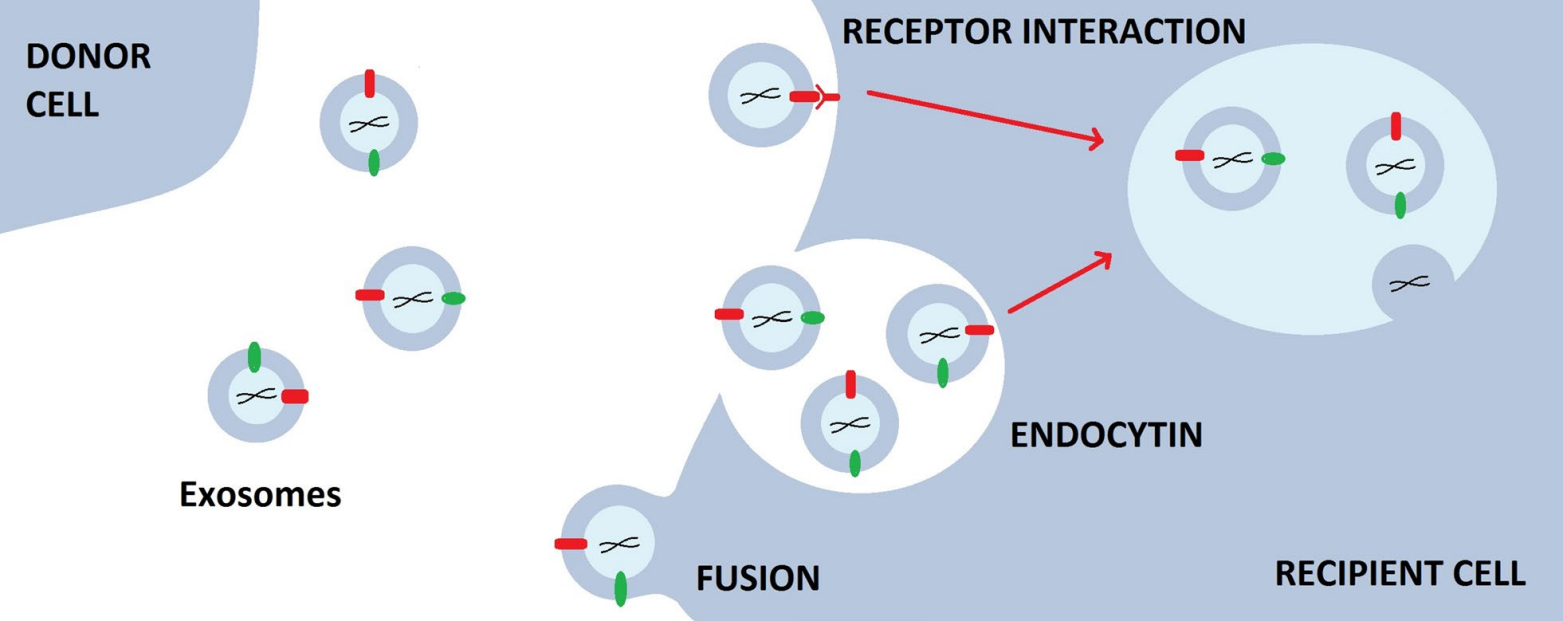
Exosome uptake

According to current studies, exosome uptake can be defined as the process whereby exosome signals are transferred to the recipient cell by a three-step mechanism; receptor interaction, membrane fusion, and endocytosis/phagocytosis (Fig. 2). Many studies also suggest that the primary method of exosome uptake is internalization which depends on cell type and exosomal surface proteins. Exosome uptake by a recipient cell is cell-specific, for which the interaction of surface molecules between specific cells and exosomes is critical for recipient cell targeting and adhesion [13].

### Derived exosomes

Exosomes have different roles depending on their progenitor cell. A significant amount of research has been carried out in an attempt to identify the roles of varied derived exosomes.

#### Macrophage-derived exosomes

Macrophages have been known for their phagocytic capability in the body’s immune system since they were discovered over 130 years ago [14]. They play a crucial role in the prevention of the progression of many diseases as they recognize and eliminate pathogenic microbial products and tumor cells [15]. Some studies have shown that they play an important role not only as an inflammatory component of cardiac injury but also as a central regulator in injury and repair [16].

Recent studies have been focused on determining the role of exosomes that derive from macrophages. The results show that integrin ICAM-1/LFA-1 interaction implicated uptake of macrophage exosomes in the unbiblical vein endothelial cell, and the c-type lectin receptor mediate the process of uptake in hCMEC/D3 cells. Furthermore, the study found that macrophage exosomes can penetrate the BBB (blood–brain barrier) to brain parenchyma in healthy mice, it even can enter the brain independently without the help from brain infiltrating immune cells and that the increased accumulation in the inflamed brain was due to an enhanced exosome–brain endothelium interaction [1].

#### Rhabdomyosarcoma (RMS)-derived exosomes

Rhabdomyosarcoma (RMS) is classified as the most common soft tissue sarcoma (STS) in children and adolescents and is a form of tumor showing distinctive traits of skeletal muscle lineage [17]. This occurs not only in children, but also in median age and older patients. RMS is included in the family of so called “small round blue tumor cell” that infiltrate bone marrow (BM) and are sometimes diagnosed as an acute leukemia cell on BM smears. RMS can be divided into two histological subtypes: embryonal rhabdomyosarcoma (ERMS) and alveolar rhabdomyosarcoma (ARMS). ARMS is associated with more aggressive behavior which has a worse prognosis than ERMS [18].

Rhabdomyosarcoma (RMS) cells have been detected to secrete exosomes. These exosomes can carry specific miRNA relevant to the cancer signaling network. Researchers have found that RMS-derived exosomes can enhance the proliferation of human recipient fibroblasts and recipient RMS cells. The exosomes can induce migration and invasion of normal human fibroblasts and also promote angiogenesis. Another study also identified a role for RMS-derived exosomes in facilitating host cell infiltration into matrigel plugs, which again plays a role in promoting invasion and angiogenesis during RMS tumor progression [19].

#### Metastatic cancer cell-derived exosomes

Exosomes have been found to exert many influences over tumor properties, such as growth, angiogenesis, invasion and metastasis, and they promote cell motility. Hence, on the whole, they promote cell formation, adhesion, and cell polarity [20].

Those exosomes that derive from cancer cells with aggressive propensities can differentially affect the properties of endothelial cells and also affect morphological and functional properties of the metastatic tumor. Exosomes were used as a mediator of intracellular communication by exporting thrombin. In another way, the exosomes activate the RhoA/Rock pathway in recipient cell [21]. These exosomes also manipulate host stromal responses to generating a protumorigenic or antitumorigenic milieu [22].

#### Malignant mesothelioma (MM) cell-derived exosomes

Malignant mesothelioma that originates from the deregulated cellular proliferation of the mesoderm tissue lining the chest cavity, heart, lungs, the abdominal cavity, and the intra-abdominal organs, is a rare type of cancer [23]. This cancer occurs in the mesothelial lining of pleural or peritoneal cavities and can be defined as an asbestos-related aggressive tumor with poor prognosis. Due to its low incidence, resistance to most chemotherapies, and the complexity of the tumor anatomy, malignant mesothelioma is classified as a deadly and clinically challenging disease to treat. The overall survival time for patients of this cancer is less than 1 year for pleural tumor, and the 5-year survival rate is less than 15% for peritoneal mesothelioma [24].

Exosome that derive from Human malignant mesothelioma cells have been shown to have an immunoregulatory function during cancer progression. They can regulate recipient cells of the tumor microenvironment and contain factors associated with metastasis. Researchers have identified a total of 111 proteins associated with immunoregulation in MM-derived exosomes of which 26 were identified in mEXOS, including oncostatin-M receptor (OSMR), multidrug resistance-associated protein 1 (ABCC1), and the SUMO-1 activating receptor, SAE1. OSMR is a multifunctional cell surface cytokine receptor, which induces several pro-malignant effects, including a pro-angiogenic phenotype and increased cell migration and invasiveness [25].

#### Osteoclast-derived exosomes

Osteoclasts that derive from hematopoietic/monocytic lineage are large multinucleated cells specialized in bone resorption, which is a physiological process regulated by the tight coupling between bone-resorbing osteoclasts and the primary osteogenesis cells (osteoblasts) and are also involved in bone remodeling [26]. Osteoclasts differentiate from myeloid precursors influenced by the cytokines macrophage colony stimulating factor (MCSF) and receptor activator of NF-κB ligand (RANKL), and supplied by osteoblasts and/or osteocytes. The receptor for RANKL, known as osteoprotegerin (OPG) is produced by the osteoblast lineage that tampers the osteoclast differentiation. Osteoclasts polarize the secretion of proteolytic enzyme and acid, which cause bone degradation. They also hydrolyze and solubilize the organic and inorganic components in bone [27].

During the process of osteoclastogenesis, exosomes are secreted that have the role of transfering miR-214 to osteoblasts to inhibit their activity. Inhibition of exosome formation and secretion from osteoclasts attenuate the effect of osteoclasts on osteoblasts. In general, exosomes express cell recognition molecules on their surfaces that facilitate selective targeting and uptake by recipient cells. In this regard, miR-214-containing exosomes act as a coupling inhibitor to negatively regulate osteoblasts. Among the coupling inhibitors, osteoclast derived ephrinA2, whether anchored onto osteoclast membranes or in soluble form, acts on osteoblasts through its Eph receptor [28].

#### Pancreatic cancer cell (PCC)-derived exosomes

Pancreatic cancer is often triggered by poor lifestyles choices including cigarette smoking, increased body mass index, heavy alcohol consumption, and also may be caused by a family history of pancreatic cancer [29]. Pancreatic cancer is particularly difficult to manage as it often has no symptoms at early stages leading to challenges in early diagnosis, and it has a rapid progression and limited response to conventional therapy. Pancreatic cancer cells (PCCs) also secrete the exosomes for cell communication [30].

By secreting exosomes, cancer cells reprogram cells in the tumor microenvironment and at distant sites to support the progression of cancer. PCC-derived exosomes therefore increase proliferation and migration of cancer cells. Moreover, PCC-derived exosomes have been proven induce fibrosis-related gene expression including induced activation and profibrogenic activities in pancreatic stellate cells [31].

#### Bronchial fibroblast-derived exosomes

Fibroblast cells are building blocks of connective tissue and create the extracellular matrix, which can be classified that the adventitial fibroblast that surrounds blood vessels and the interstitial fibroblast that is not closely associated with any specific structure [32]. Fibroblasts play a key role in airway inflammation by expressing an array of cytokines and adhesion molecules and also in infiltration and activation of eosinophils and other leukocytes. They can be classified as the most abundant cell type within the lung interstitium and can be transformed into the myofibroblast phenotype, which expresses contractile proteins and has an increased capacity for collagen deposition, in a process regulated by cytokines, growth factors and matrix components [33].

Haj-Saleem et al. [34] have determined the role of bronchial fibroblast-derived exosomes. Their research showed that bronchial fibroblasts secreted exosomes, which are internalized by bronchial epithelial cells. Exosomes derived from fibroblasts of severe asthmatic subjects showed a lower level of TGF-b2 and significantly increased the epithelial cell proliferation of both healthy and severe asthmatic subjects compared to healthy control exosomes. Overexpression of TGF-b2 in the fibroblasts of severe asthmatics induced enhanced TGF-b2 in exosomes leading to a reduced proliferation of epithelial cells, whereas the reduction of TGF-b2 enhanced epithelial cell proliferation.

In short, it can be concluded that bronchial fibroblast-derived exosomes play a role as a messenger for the increased proliferation of airway epithelium in cases of severe asthma. Exosomes derived from fibroblasts of severe eosinophilic asthmatics can contribute to airway remodeling, at least in part, by modulating epithelial cell [34].

#### Mesenchymal stem cell (MSC)-derived exosomes

Arnold Caplan coined the term “Mesenchymal stem cell (MSC)” in the early 1990s, which were described for the first time in 1970s by Alexander Friedenstein as a population of bone marrow stromal cells capable of mesodermal differentiation and trophic support of hematopoiesis [35]. MSC can be harvested from several adult tissues and it can constitute a heterogeneous subset of stromal regenerative cells [36].

Similar to exosomes in general, MSC-derived exosomes carry complex cargo, including nucleic acids, proteins, and lipids. MSC-derived exosomes are highly enriched in biologically active molecules, such as proteins and RNAs, and are therefore well equipped to maintain homeostasis within the tissue and respond to external stimuli. Another study also showed that MSC-derived exosomes potentially have therapeutic properties in liver disease. In myocardial I/R injury, MSC-derived exosomes exert protective effects against myocardial I/R injury possibly through several mechanisms, such as anti-apoptosis, cardiac regeneration, anti-cardiac remodeling, anti-inflammatory effects, neovascularization and anti-vascular remodeling [37].

### Isolation of exosomes

**Fig. 3:**
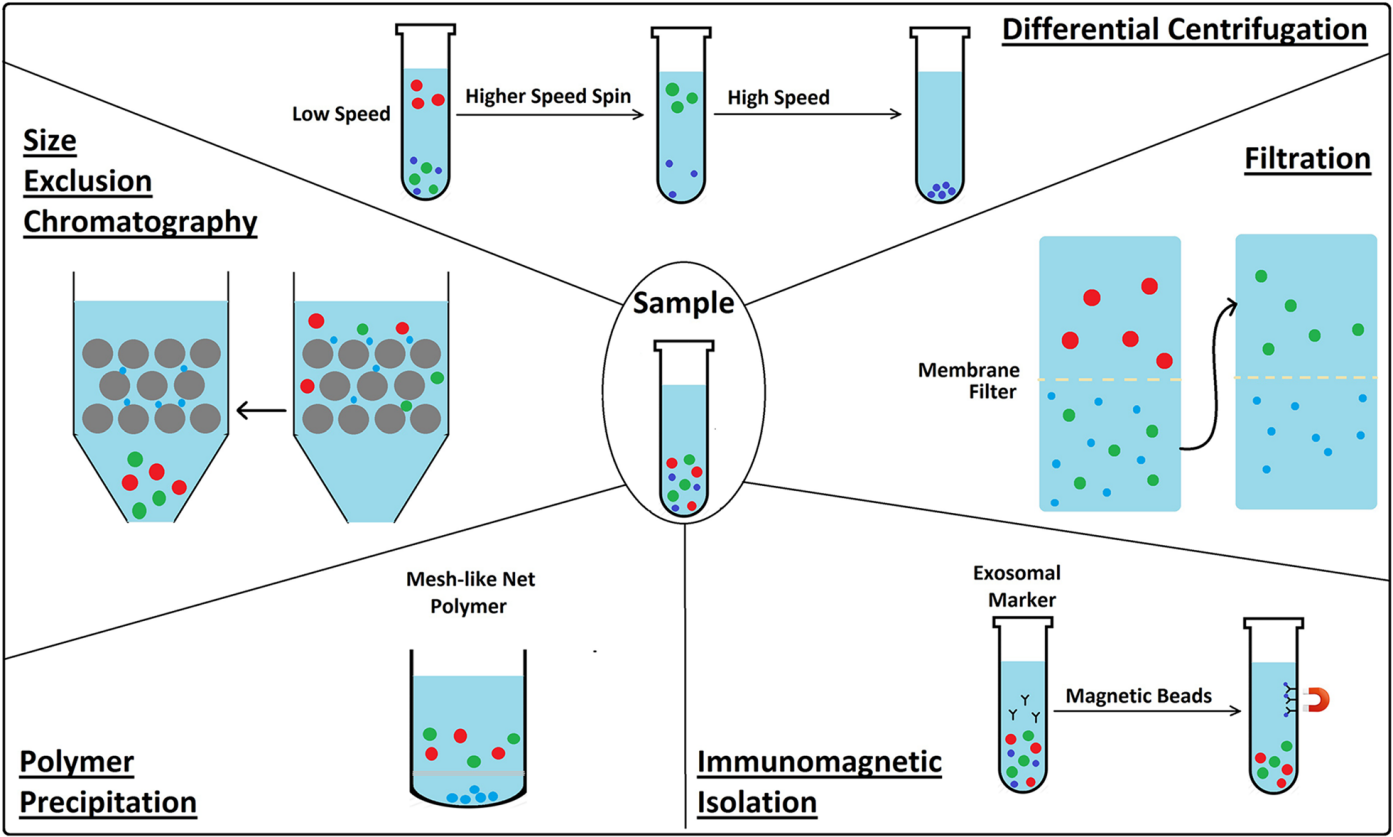
Schematic diagram of exosome isolation

Currently, exosomes are isolated by differential centrifugation, filtration, size-exclusion chromatography, and polymer precipitation (Fig. 3). To obtain ultrapure exosomes or isolation of the potential subpopulation of exosomes, an immunomagnetic isolation strategy can be applied by targeting exosomal markers [38].

#### Differential centrifugation

The most widely used method for exosome isolation is differential centrifugation [39], it is adopted as a reliable technique for isolating exosomes from biological fluids [40]. The method consists of several steps, including (1) a low-speed centrifugation to remove cells and apoptotic debris, (2) a higher speed spin to eliminate larger vesicles and finally, (3) high-speed centrifugation to precipitate exosomes. However, the efficiency of the method is lower when viscous biological fluids such as plasma and serum are used for analysis [41]. The quantity of collected EVs is affected by centrifuging speed and time, so these parameters must be optimized for each rotor type [42].

#### Filtration

Commercial membrane filters or polycarbonate can be used to isolate cells and large EVs in biological samples. Filtration methods are often combined with ultracentrifugation, where membranes are used to sieve cells and large EVs, after which separation of exosomes from proteins is achieved via ultracentrifugation. In order to eliminate the need for ultracentrifugation, a number of research groups have explored commercial ultrafiltration as a means to separate exosomes from protein contaminants [43]. Exosomes can be separated from other soluble proteins and aggregates using matrices with defined molecular weight or size exclusion limits. These vesicles can be selectively isolated based on a molecular weight greater than 2 million Daltons, followed by isolation with a diameter less than 200 nm. This allows the separation of smaller aggregates and soluble components from exosomes [44].

#### Size-exclusion chromatography

Size-exclusion chromatography (SEC), also known as molecular sieve chromatography [45] is a method where the separation of different compounds occurs according to their size (hydrodynamic volume) measured by how efficiently they penetrate the pores of the stationary phase. Size exclusion chromatography has two basic versions. When performed using organic solvents, it is called gel permeation chromatography (GPC). The main application field of GPC is in polymer analysis. When size-exclusion chromatography is performed using aqueous solvents, it is called gel filtration [46]. As compared to the ultracentrifugation method, having highly varying EV yields (2–80%), this method is superior with 43% stable recovery of EVs, with almost complete removal of contaminating proteins. Disadvantages of this method are (1) the accessibility of the chromatography column to contamination, therefore aseptic working conditions should be ensured especially if the isolated EVs are intended for therapeutic use; (2) a large number of fractions should be collected and analysed in order to make sure complete separation of EV subtypes and contaminating proteins and (3) contrarily to the simplicity and time effectiveness of the separation protocol, post-isolation analysis of each fraction may be rather time consuming [47].

#### Polymer precipitation

Polymer precipitation is a technique used to isolate and purify polymers. The methods of polymeric precipitation are based on the formation of a mesh-like net that embeds EVs with a size ranging from 60 to 180 nm. These methods may be applied to culture media or to bodily fluids. In particular, polymeric precipitation methods may have the advantage in the detection of biomarkers in vesicles derived from small biological samples [48].

#### Immunomagnetic isolation

Exosomes are observed in most bodily fluids containing typical exosomal markers such as CD9, CD63, and CD81. Potential subpopulations of exosomes can be captured by targeting these markers using magnetic beads. Magnetic beads are versatile tools for exosome isolation and downstream analysis [49]. Immunomagnetic isolation uses the antibody-labeled magnetic beads which can specifically capture exosomes and the magnetic field will separate the captured exosome from other substances [50].

## Exosome as a therapeutic delivery system

An exosome-based delivery system has particular benefits such as specificity, safety, and stability. By their homing characteristic, exosomes can deliver their cargo to specific targets over a long distance. Exosomes can also be used to deliver interfering RNA (siRNA) or pharmaceutically active substances [51]. As exosomes are small and native to animals, they are able to avoid phagocytosis, fuse with the cell membrane, and bypass the engulfment by lysosomes. The fact that exosomes are a natural product of the body results in a low immune response [52]. Exosome also can exhibit increased stability in the blood that allows them to travel long distances within the body under both physiological and pathological conditions. Furthermore, exosomes have a hydrophilic core, which makes them suitable to host water-soluble drugs [53]. Several methods for exosome loading have been suggested to date, which can be classified into two different strategies, cargo loading after isolation and cargo loading during formation [54].

For cargo loading after isolation, a few loading procedures have been reported (Table 1). One of the methods is electroporation. By applying an electric field to a suspension of exosomes (or cells) and the therapeutic cargo of choice, pores are created into the lipid bilayer membrane, thereby facilitating the movement of cargo into the lumen of the exosomes. Simple incubation of exosomes with the cargo was also used as one of the methods of loading exosomes. Curcumin was efficiently loaded into exosomes after only 5 min of incubation at 22 °C and was shown to mediate significant anti-inflammatory effects in several disease models such as brain inflammation, autoimmune disease and brain tumors [55, 56]. Another method to load cargo into exosomes is sonication. A drug-exosome mixture was sonicated for six cycles of 30 s on/off for a total of 3 min with 2 min cooling period, which resulted in effective drug loading into exosomes. Taking the size, the zeta potential and the quantity of drug loading into account, there are no significant changes in the structure and content of exosomal membranes after sonication [53]. The extensive reformation and reshaping of exosomes upon sonication and extrusion procedures enabled catalase diffusion across the relatively tight and highly structured lipid bilayers and resulted in the high loading efficiency of exosomal carriers (20–26% loading capacity) [57].

**Table 1:** Type of cargo and loading method [58–66]

Type of exosome	Cargo	Loading method	References
RAW 264,7 exosome	Catalase enzyme	Incubation	[58]
		Freeze thaw cycle	
		Sonication	
		Extrusion	
Rabies viral glycoprotein (RVG) exosome	Opioid receptor Mu siRNA	Incubation	[59]
EL-4 and RAW 264,7 exosome	Curcumin	Incubation & simple mixing	[60]
B16 melanoma exosome	Superparamagnetic iron oxide nanoparticles	Electroporation	[61]
LNCaP and PC-3 exosome	Paclitaxel	Incubation	[62]
RAW 264,7 exosome	Doxorubicin and paclitaxel	Incubation	[63]
		Electroporation	
		Sonication	
iRGD exosome	Doxorubicin	Electroporation	[64]
Milk-derived exosome	Paclitaxel	Simple mixing	[65]
HEK293T exosome	DNA	Electroporation	[66]

## Modified exosome for drug delivery

Nowadays, nanoscale drug delivery systems have attained considerable prominence. Various nano-based drug formulations have been used to improve the therapeutic efficacy of chemical and biomolecular drugs. Since the discovery that exosomes function as intercellular communication tools that transfer their cargo to recipient cells, they have garnered considerable research attention [67].

The ideal drug delivery system should be capable of site-specific delivery of incorporated therapeutics, avoid recognition and premature degradation by the body’s immune defenses and controlled release of cargo molecules upon selective stimuli. Exosomes have the ability to deliver endogenous biological cargo, such as small RNAs, mRNAs, and proteins across cells. Exosomes have shown many advantages in terms of biocompatibility and reduced clearance rates in view of their natural origin in comparison with other DDS. Moreover, they show little long-term accumulation in any organ or tissue, with concurrent low systemic toxicity, and also facilitated cellular uptake [68].

### Surface modified exosomes for cerebral ischemia therapy

Cerebral ischemic can be classified as the condition whereby impaired blood flow does not allow sufficient deliver of oxygen and glucose, leading to energy depletion, over-activation of glutamate receptors and release of excess glutamate, an increase of intracellular calcium, loss of membrane potential and cell depolarization, and, eventually, cell death [69].

**Fig. 4:**
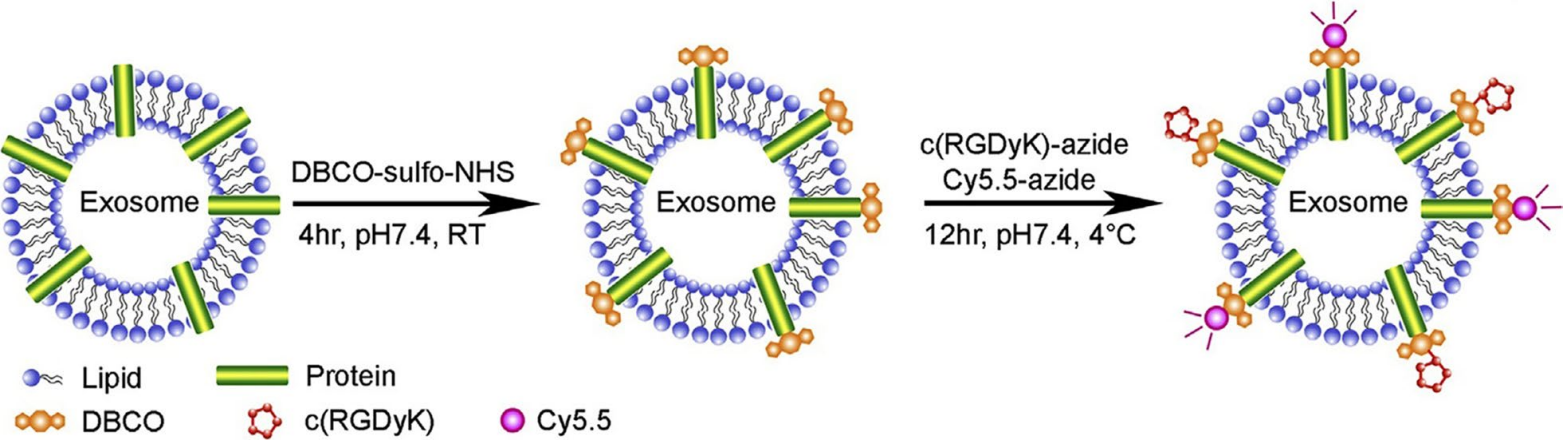
Exosomes modified with DBCO, c(RGDyK), Cy5,5 [70]

Using exosomes as nanocarriers is one of many methods for cerebral ischemic therapy. The targeting ability of exosomes can be improved through appropriate surface modification. Tian et al. has proposed a simple, rapid and efficient method to conjugate functional ligands onto exosomal surfaces using bioorthogonal copper-free azide-alkyne cycloaddition. The cyclo(Arg-Gly-Asp-D-Tyr-Lys) peptide [c(RGDyK)], which exhibits high affinity to integrin αvβ3 in reactive cerebral vascular endothelial cells after ischemia specifically, was conjugated on the mesenchymal stromal cell (MSC)-derived exosome surface. Furthermore, curcumin, a natural polyphenol from *Curcuma longa*, was loaded onto the cRGD-Exo (Fig. 4). The result shows that modified exosome molecules showed greater accumulation in ischemic brain as compared to unmodified exosome molecules [70].

### Exosomes combine with pH-sensitive fusogenic peptide and cationic lipid for cytosolic delivery

GALA is composed of repeating sequences of Glu-Ala-Leu-Ala and has an amphiphilic structure, so is a pH-sensitive peptide. Endosomal pH degradation can decrease the negative charge of Glu residues and convert the structure from random to helical. GALA has been widely used to improve transfection efficiency [71]. Different from GALA, cationic lipids are considered to affect various properties of DNA–cationic liposome complexes and play a very important role in efficient gene transfer [72].

**Fig. 5:**
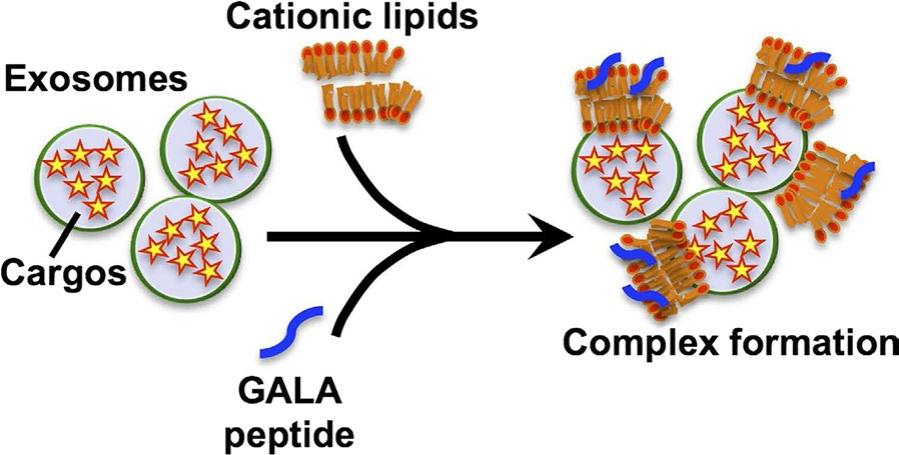
Exosome modified with GALA peptide and cationic lipid [73]

In 2014, Nakase and Futaki combined GALA, cationic lipid, and exosome for cytosolic delivery (Fig. 5). They selected this combination as a high amount of exosomes in bodily fluids induces competition for their cellular uptake by endocytosis, and as a result the cellular uptake efficiency of exosomal vehicles for intracellular delivery is considered insufficient for therapeutic treatment. This research group successfully achieved the cytosolic delivery of peptides and proteins using the GALA peptide in a combined treatment with cationic lipids. The GALA peptide was designed to mimic viral fusion protein sequences that interact with cell membranes to mediate the escape of the viral genes from acidic endosomes [73].

### Exosome engineering with PEG and AA for pulmonary metastasis

Lung cancer is one of the deadly cancers and is a leading cause of cancer mortality worldwide. Lung carcinomas are divided into two parts; small-cell lung carcinoma (SCLC) and non-small-cell lung carcinoma (NSCLC), where NSCLC represents the majority (> 85%) cases. NSCLC is further subclassified as adenocarcinoma (50%), squamous cell carcinoma (~ 40%) and large cell carcinoma (~ 10%). Lung cancer in NSCLC is difficult to treat effectively as the pathology of NSCSC is still unclear [74].

Recent research has focused on attempting to overcome NSCLC by using nanocarrier technology. In this research, paclitaxel-loaded exosomes were modified with PEG and AA (ligand) to improve their circulation time in the blood and allow to target pulmonary metastases. By using this modified exosome, the drug can selectively deliver to target cancer cells and also can increase the survival rate of lung cancer patients [75].

### Engineering exosomes by fusion with liposome

A liposome is a structure that consists of single or multiple concentric lipid bilayers encapsulating an aqueous compartment, which were found by Alec D Bangham in the 1960s at the Babraham Institute, University of Cambridge [76]. Liposomes have suitable properties for drug delivery as it can enhance the therapeutic outcomes for pharmaceuticals, biopharmaceuticals, and vaccines. Due to their unique structure, the liposomes are able to incorporate drugs both within their aqueous core and their lipid bilayers [77].

**Fig. 6:**
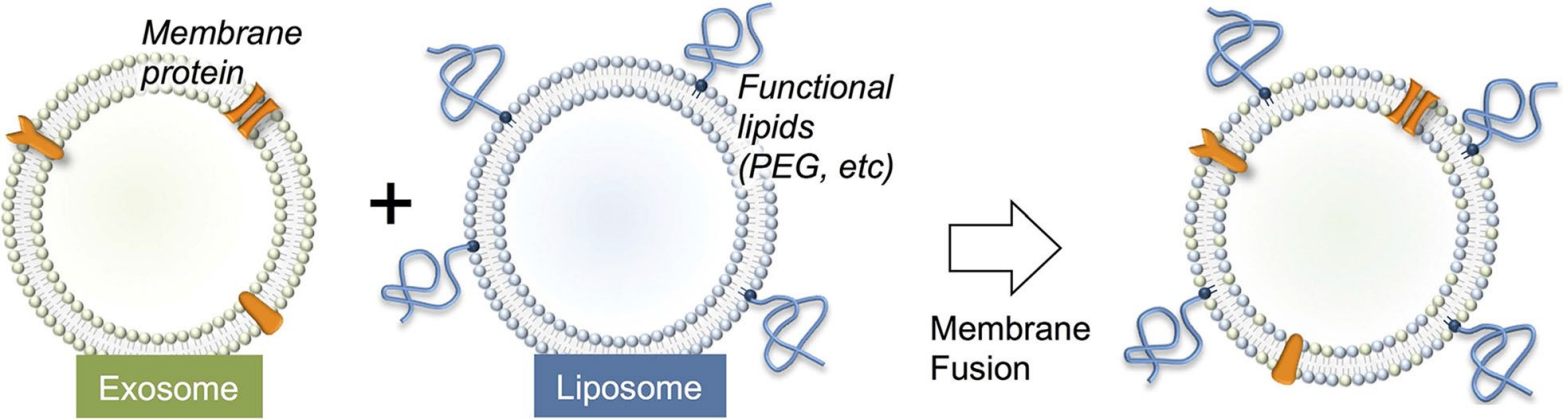
Exosomes fusion with liposome [78]

To increase the delivery function of the exosomes, Sato et al. attempted to fuse the membranes of exosomes with liposomes using the freeze–thaw method. This research aims to optimize the exosome surface in order to decrease its immunogenicity and increase its colloidal stability and improve the half-life of exosomes in blood. The result of this research suggests that this represents a new method for hybrid exosomes as novel biological nanotransporters (bio-nanotransporter). These fusion exosomes can be used to transport exogenous hydrophobic lipid and also hydrophilic cargoes by membrane fusion method to recipient cells [78] (Fig. 6).

### Exosome-coated metal–organic framework nanoparticle

Over the last two decades, metal–organic frameworks (MOFs) have become popular among researchers due to their intriguing architectures, high crystallinity, exceptional porosity and diverse modularity that can be employed in various fields [79]. MOFs are a kind of organic–inorganic hybrid compound with one-, two-, or three-dimensional (1D, 2D, 3D) structural topologies consisting of inorganic metal ions/clusters and organic ligands [80].

MOFs have many potential factors that may be used in nanomedicine, such as structural and chemical diversity, their high loading capacity, and intrinsic biodegradability [81]. Many more conventional drug delivery vehicles such as micelles, liposomes, and dendrimers have difficulties in the control of drug release. On the other hand, MOF nanoparticles have a high loading capacity and controlled drug-release properties [82].

**Fig. 7:**
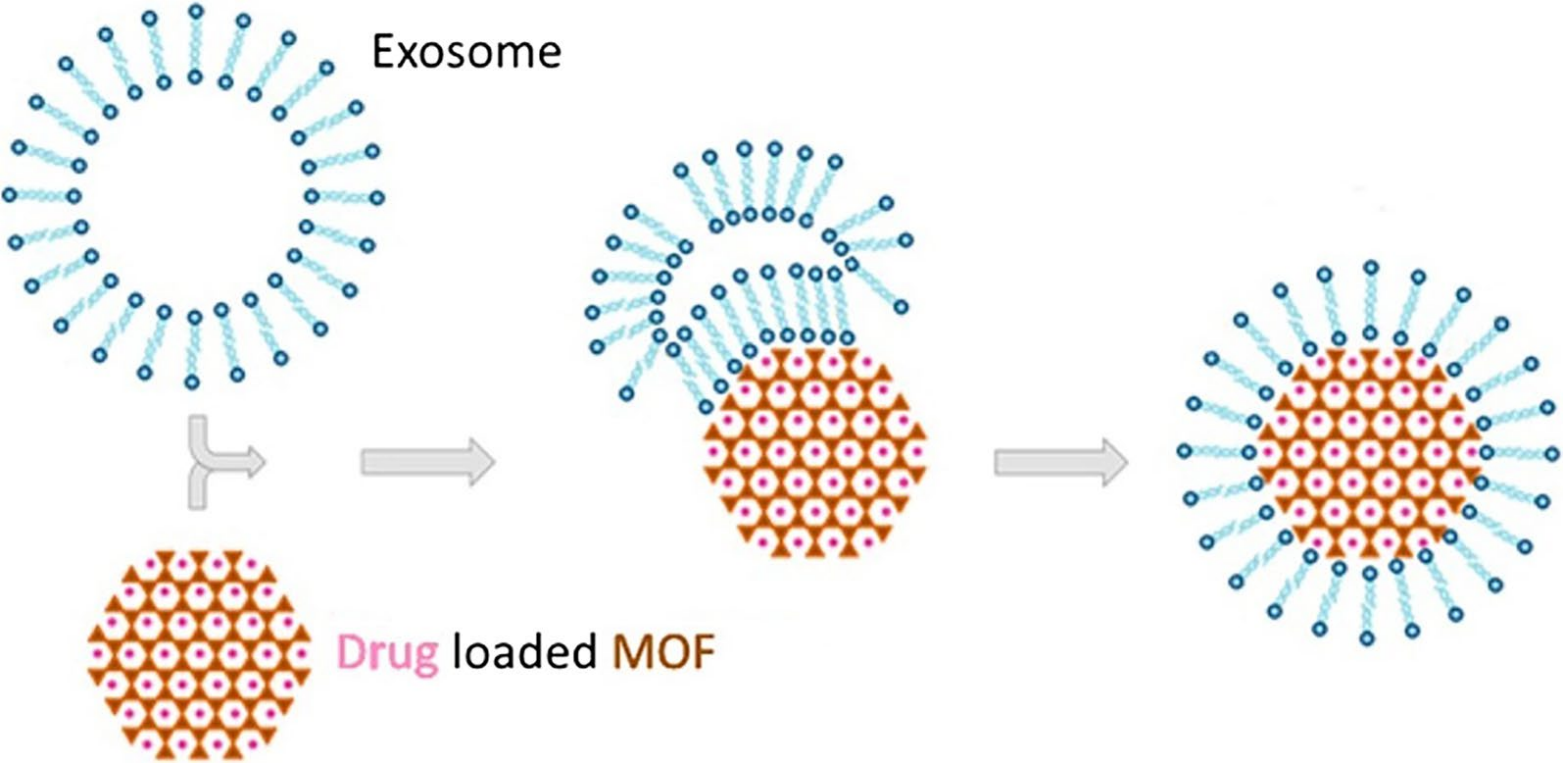
Exosome-coated metal–organic framework [83]

In 2017, Illes and co-workers reported the use of MOFs for drug delivery. They combined MOF with exosome as the drug carrier. The result shows that exosome-coated MOF NPs are a smart and efficient drug delivery system with an “onboard-trigger”. They combine the features of MOF NPs and exosomes facilitating simple and efficient loading and sealing of cargos (Fig. 7). Moreover, it shows great delivery ability with no premature leakage. Intracellular cargo release is possibly mediated by a combination of the endogenous exosomal release mechanism and degradation of the nanocarrier, which decomposes into substances that are naturally present in the body [83].

## Discussion

Nowadays, the study of exosomes has become a popular in molecular technology research. The precise roles of exosomes produced by a variety of cells are still unclear and need further research. Accordingly, many studies have focused on revealing the role of exosomes that were derived from specific cells, such as macrophages, rhabdomyosarcoma (RMS) cell, metastatic cancer cell, malignant mesothelioma (MM) cell, osteoclast, pancreatic cancer cell (PCC), bronchial fibroblast and mesenchymal stem cell.

RMS-derived exosomes were discovered to deliver miRNA to induce migration and invasion of human fibroblast and promote angiogenesis. On other hand, exosomes that derived from osteoclasts delivered specific miRNA that acts as a coupling inhibitor to negatively regulate osteoblast. Exosomes that derived from bronchial fibroblast cells play a role as messenger to increase proliferation of airway epithelium in severe asthma that can induce airway remodeling. Exosomes that were produced from cancer cells have different roles depending on their progenitor cancer cell. Metastatic cancer cell-derived exosomes export thrombin and activate the RhoA/Rock pathway in recipient cell, while PCC-derived exosomes play a role in increasing proliferation and migration and induce activation and profibrogenic in pancreatic stellate cell. Other exosomes whose roles have been identified are MM-derived exosomes, macrophage exosomes, and MSC exosomes. Macrophage exosomes were discovered to play an important role in the body’s immune system. MM-exosomes were identified regulate recipient cells within the tumor microenvironment which induce pro-malignant effects such as pro-angiogenesis and increasing angiogenesis and invasiveness, while MSC-exosomes play role in maintaining homeostasis, responding to external stimuli and exerting a protective effect against myocardial injury.

Using derived exosomes for drug delivery has great advantages as exosomes that derived from a specific progenitor cell will deliver cargos to the specific target cell. As for the delivery properties, the ideal derived exosome is the macrophage exosome. In our review, we have explained that macrophage exosomes have the ability to enhance the BBB, even without any modifications on their surface. This exosome is suitable for drug delivery for brain treatment since it has better ability to cross the BBB compared to other nanotechnology. In another hand, Metastatic cancer cell-derived exosomes, MM-exosomes, and PCC-exosomes are useful properties for cancer treatment as these exosomes have an important role in cancer cell maintenance that also affects cancer growth. Others derived exosomes have the same role as exosome in general that can be used for targeting the specific target for treatment, such as RMS-exosomes for targeting fibroblast cells, osteoclast exosome for targeting osteoblast cells and bronchial fibroblast-exosome for targeting epithelial cells.

Not only revealing the roles of the exosomes, much researcher has been carried out on using them to deliver cargo to specific target cell. It has also been shown that their delivery ability can be increased via modification of their surface. Tian et al. modified the exosomes with DBCO, c(RGDyK), Cy5,5 in order to deliver curcumin to the ischemic sites in the brain. As exosomes have low cytosolic release efficiency of the encapsulated molecule inside the cell, Nakase and Futaki in 2014 combined exosomes with GALA peptides and cationic lipids to enhance the cellular uptake. Another innovation is exosome fusion with liposome. Exosomes and liposomes each have their own advantageous delivery properties. Sato et al. combined these two particles in order to decrease immunogenicity and increase the colloidal stability. In 2017, Illes et al. and Kim et al. reported modification with a different purpose. Illes et al. combined exosomes with metal organic frame nanoparticles to make them more efficient in delivering their cargo and increasing their therapeutic efficacy. Kim et al. used PEG and ligand AA to be combined with exosomes to increase the circulation time and target pulmonary metastasis.

In summary, exosomes are one of the ideal and competitive nanocarriers for drug delivery even with or without being modified. Natural derived exosomes have their own role in cell–cell communication, which imparts them with useful as delivery properties. However, development of modified exosomes also has a promising future in drug delivery research. However, it is important to do more research to improve characterization proses and standardize production for either derived exosomes or modified exosomes for drug delivery systems.
